# Supplementary material for: Forest carbon stocks increase with higher dominance of ectomycorrhizal trees in high latitude forests
Source: Nat Commun. 2024 Jul 16;15:5959. doi: 10.1038/s41467-024-50423-9 (PMC11251171; doi:10.1038/s41467-024-50423-9)
Supplement: Supplementary file 1 — Supplementary Information [file 41467_2024_50423_MOESM1_ESM.pdf]

Supplementary Material for

Forest carbon stocks increase with higher dominance of ectomycorrhizal trees in high latitude forests

Guoyong Yan[1], Chunnan Fan[2], Junqiang Zheng[3], Guancheng Liu[1], Jinghua Yu[4], Zhongling Guo[2],Wei Cao[4], Lihua Wang[4], Wenjie Wang[5], Qingfan Meng[2], Junhui Zhang[1], Yan Li[2], Jinping Zheng[2], Xiaoyang Cui[5], Xiaochun Wang[5], Lijian Xu[6], Yan Sun[6], Zhi Zhang[7], Xiao-Tao Lü[4], Ying Zhang[4], Rongjiu Shi[4], Guangyou Hao[4], Yue Feng[4], Jinsheng He[8], Qinggui Wang[1*], Yajuan Xing[1,6*], Shijie Han[1,3,4*]

[1] School of Life Sciences, Qufu Normal University, Qufu, 273165, China

[2] School of Forestry, Beihua University, Jilin, 132013, China

[3] School of Life Sciences, Henan University, Kaifeng, 475004, China

[4] Institute of Applied Ecology, Chinese Academy of Sciences, Shenyang, 110016, China

[5] School of Forestry, Northeast Forestry University, Harbin, 150040, China

[6] College of Modern Agriculture and Ecological Environment, Heilongjiang University, Harbin, 150080, China

[7] College of Ecology, Lishui University, Lishui, 323000, China

[8] College of Urban and Environmental Sciences, Peking University, Beijing, 100871, China

* To whom correspondence may be addressed.

E-mail: qgwang1970@163.com (Q.W.); xingyajuan@163.com (Y.X.); hansj@iae.ac.cn (S.H.)

Tel.: +86 357 7038967. Fax: +86 357 7037003.

Supplementary Material

Supplementary Methods

Fungal community measurements

Ten soil samples were randomly collected from each plot using a soil auger with a 5 cm diameter, focusing on the top 0-10 cm soil layer. After collection, all soil samples were thoroughly mixed and homogenized to produce a single composite soil sample per plot. Each fresh soil sample was then sieved through a 2 mm mesh sieve and divided into two subsamples. One of these subsamples was promptly stored in dry ice and transported to the laboratory, where it was preserved at -80°C for subsequent analysis.

Soil DNA was extracted from 0.5 g of fresh soil using the OMEGA Soil DNA Kit (Omega Bio-Tek, Norcross, GA, USA) following the manufacturer's protocol. The extracted DNA was quantified using a NanoDrop 2000 spectrophotometer (Thermo Fisher Scientific Inc., USA), and its quality was assessed via 1.2% agarose gel electrophoresis. PCR amplification of the ITS1 region of the fungal 18S rRNA gene was carried out using the primers ITS1F (5′-GGAAGTAAAAGTCGTAACAAGG-3′) and ITS2R (5′-GCTGCGTTCTTCATCGATGC-3′). Sample-specific seven-bp barcodes were incorporated into the primers to enable multiplex sequencing. PCR amplicons were purified using Vazyme VAHTSTM DNA Clean Beads (Vazyme, Nanjing, China) and quantified with the Quant-iT PicoGreen dsDNA Assay Kit (Invitrogen, Carlsbad, CA, USA) using a Microplate reader (BioTek, FLx800). After quantification, the

purified amplicons were pooled at equimolar concentrations and subjected to paired-end sequencing (2 × 250) using the MiSeq Reagent Kit V3 (600 cycles) on an Illumina MiSeq platform (Illumina Inc., San Diego, CA, USA) at Shanghai Personal Biotechnology Co., Ltd (China).

The raw FASTQ files underwent quality filtering using QIIME2 (Bolyen et al., 2019). Sequences containing ambiguous bases, more than one mismatch to the primers, homopolymers longer than 13 bp for fungi (Soonvald et al., 2019), and those lacking a minimum overlap of 50 bp were excluded. High-quality sequences were subsequently clustered at a 97% similarity cut-off to generate operational taxonomic units (OTUs) using Vsearch (v2.13.4). Chimeric sequences were identified and removed using UCHIME (version 4.2.40, http://drive5.com/usearch/manual/uchime algo.html). Taxonomy assignment of fungal OTUs relied on the Unite database (Nilsson et al., 2019), with sequences not assigned to the kingdom Fungi being discarded. FUNGuild (version 1.0) was then employed to analyze the functional groups of fungi (Nguyen et al., 2016). Low abundance OTUs (≤ 10 sequences across all samples) were removed from the fungal datasets to mitigate PCR or sequencing artifacts (Oliver et al., 2015). The average fungal OTU richness of each plot in the study was 127,541, with over half (78,126) assigned to Ectomycorrhizal (EcM) and Saprotrophic (SAP) fungi based on FUNGuild classifications. Alpha-diversity indexes, including Shannon, Chao1, and Simpson indexes, were estimated for EcM and SAP fungi using the MOTHUR software (version 1.30.1).

Table S1| **Summary of mixed-effects models (LMMs) predicting tree carbon stocks, soil carbon stocks, and forest carbon stocks using the raw data.** The following fixed effects were tested in LMMs: ectomycorrhizal tree dominance (EcM), Tree species richness (SR), Succession (SUS), Climate (CL), elevation, slope, and the interactions EcM × SR, EcM × SUS, and EcM × CL. Fixed terms were fitted sequentially (Wald tests) as indicated in the table. Random terms were ecoregion. Explanations: df, numerator degrees of freedom; ddf, denominator degrees of freedom; F value indicates F ratios, p indicates the p-value of the significance test, and bold values indicate *p*<0.05. Upward-pointing arrows indicate positive effects, downward-pointing arrows indicate negative effects. The LMG shows the percentage of variance explained by each model predictor within the entire variance explained.

Predicted variable	Predictor	df	ddf	F	P-value	LMG (%)
Tree carbon stocks	EcM tree dominance (EcM)	1	4458.30	117.52	**<0.001** ↑	14.25
	Tree species richness (SR)	1	4242.20	188.47	**<0.001** ↑	40.16
	Succession (SUS)	1	4455.30	33.32	**<0.001** ↑	9.09
	Climate (CL)	1	3353.70	14.39	<0.001 ↓	15.45
	Elevation	1	3740.90	20.93	**<0.001** ↑	1.22
	Slope	1	4447.20	33.90	**<0.001** ↓	6.48
	EcM×SR	1	4421.80	55.33	**<0.001** ↓	6.89
	EcM×SUS	1	4454.70	13.54	**<0.001** ↓	2.37
	EcM×CL	1	4398.40	5.18	**0.013** ↑	4.04
Soil carbon stocks	EcM tree dominance (EcM)	1	2008.90	12.16	**<0.001** ↑	10.57
	Tree species richness (SR)	1	2008.50	7.59	**0.006** ↑	1.21
	Succession (SUS)	1	2017.20	1.71	0.191	3.36
	Climate (CL)	1	2018.90	73.69	**<0.001** ↓	10.98
	Elevation	1	1954.70	37.71	**<0.001** ↑	24.01
	Slope	1	2022.50	1.94	0.162	10.68
	EcM×SR	1	2022.90	11.34	**<0.001** ↓	11.22
	EcM×SUS	1	2017.80	1.74	0.187	0.96
	EcM×CL	1	2024.80	16.37	**<0.001** ↑	26.97
Froest carbon stocks	EcM tree dominance (EcM)	1	2012.40	44.18	**<0.001** ↑	15.62
	Tree species richness (SR)	1	2012.10	34.24	**<0.001** ↑	8.66
	Succession (SUS)	1	2017.10	0.10	0.743	6.85
	Climate (CL)	1	2020.50	47.47	**<0.001** ↓	18.01
	Elevation	1	1966.30	59.90	**<0.001** ↑	15.74
	Slope	1	2022.20	11.23	**0.002** ↓	13.28
	EcM×SR	1	2023.60	27.23	**<0.001** ↓	10.79
	EcM×SUS	1	2017.70	0.67	0.411	0.10
	EcM×CL	1	2024.50	7.43	**0.006** ↑	10.91

Table S2| **Summary of mixed-effects models (LMMs) predicting tree carbon stocks, soil carbon stocks, and forest carbon stocks using the sampled data.** The following fixed effects were tested in LMMs: ectomycorrhizal tree dominance (EcM), Tree species richness (SR), Succession (SUS), Climate (CL), elevation, slope, and the interactions EcM × SR, EcM × SUS, and EcM × CL. Fixed terms were fitted sequentially (Wald tests) as indicated in the table. Random terms were ecoregion. Explanations: df, numerator degrees of freedom; ddf, denominator degrees of freedom; F value indicates F ratios, p indicates the p-value of the significance test, and bold values indicate $p<0.05$.

Predicted variable	Predictor	df	ddf	F	P-value
Tree carbon stocks	EcM tree dominance (EcM)	1	2247.70	47.50	**<0.001**
	Tree species richness (SR)	1	2002.00	54.73	**<0.001**
	Succession (SUS)	1	2249.00	14.39	**<0.001**
	Climate (CL)	1	1307.70	8.84	**<0.001**
	Elevation	1	1117.70	23.09	**<0.001**
	Slope	1	2248.50	15.73	**<0.001**
	EcM×SR	1	2167.20	12.98	**<0.001**
	EcM×SUS	1	2248.80	6.99	**0.008**
	EcM×CL	1	2246.70	4.52	**0.011**
Soil carbon stocks	EcM tree dominance (EcM)	1	973.76	8.68	**0.003**
	Tree species richness (SR)	1	929.45	10.81	**0.001**
	Succession (SUS)	1	969.81	0.79	0.375
	Climate (CL)	1	963.00	17.62	**<0.001**
	Elevation	1	872.14	18.76	**<0.001**
	Slope	1	967.32	0.05	0.832
	EcM×SR	1	955.67	11.22	**<0.001**
	EcM×SUS	1	968.70	1.80	0.180
	EcM×CL	1	974.99	7.22	**0.007**
Froest carbon stocks	EcM tree dominance (EcM)	1	974.41	14.37	**<0.001**
	Tree species richness (SR)	1	904.48	20.15	**<0.001**
	Succession (SUS)	1	970.69	1.39	0.238
	Climate (CL)	1	953.56	14.66	**<0.001**
	Elevation	1	833.19	31.66	**<0.001**
	Slope	1	967.79	0.97	0.324
	EcM×SR	1	942.73	14.24	**<0.001**
	EcM×SUS	1	969.45	1.64	0.200
	EcM×CL	1	974.67	8.78	**0.003**

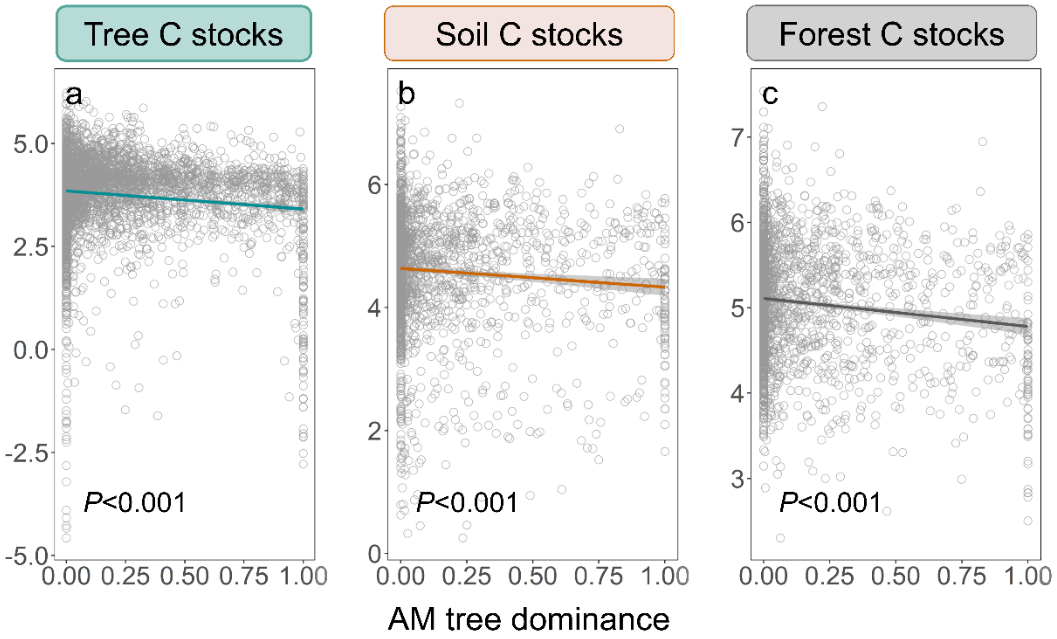

Figure S1 **Observed relationship between arbuscular mycorrhizal (AM) tree dominance and carbon stocks in forests: a, tree carbon stocks; b, soil carbon stocks; c, forest carbon stocks.** AM tree dominance is quantified as the AM proportion based on tree basal area. The solid line represents the regression fitted across all forest plots, with the solid line indicating a significant correlation ($p < 0.05$), and gray bands represent a 95% confidence interval. Each gray circle represents the data of one forest plot (tree carbon stocks, n = 4525; soil carbon stocks, n = 2035; forest carbon stocks, n = 2035).

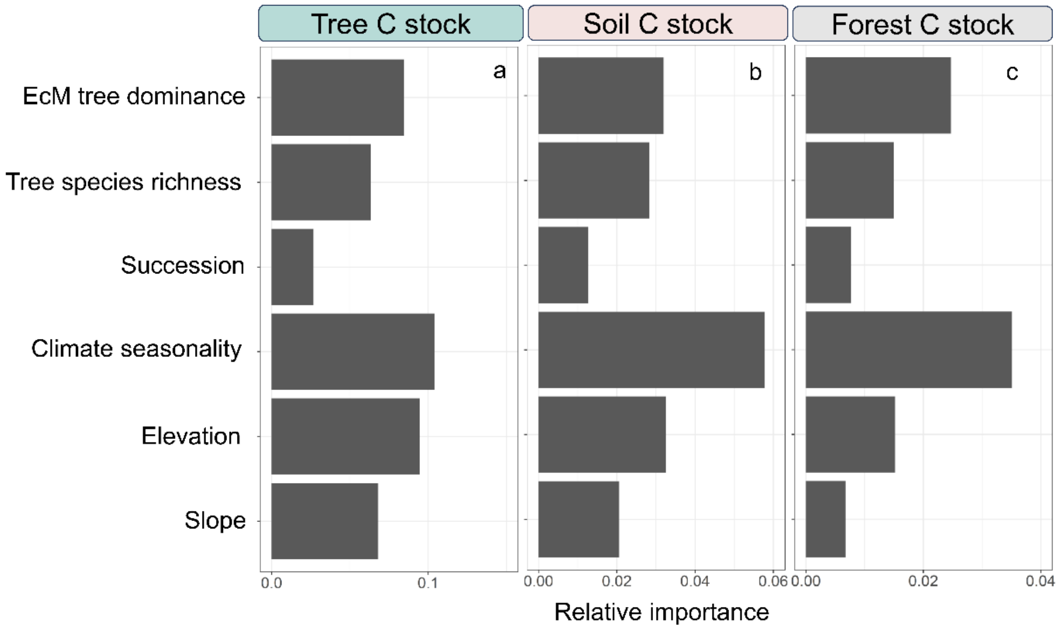

Figure S2 **Relative variable importance was determined from a random forest model explaining tree (a), soil (b), and forest (c) carbon stocks.** This importance is calculated as the mean decrease in squared error caused by each variable, rescaled to sum up to the total pseudo-R2 of the entire model. The overall explained variation (R^2) for tree, soil, and forest carbon stocks is 0.30, 0.15, and 0.17, respectively. Source data are provided in a Source Data file. EcM tree dominance, ectomycorrhizal tree dominance.

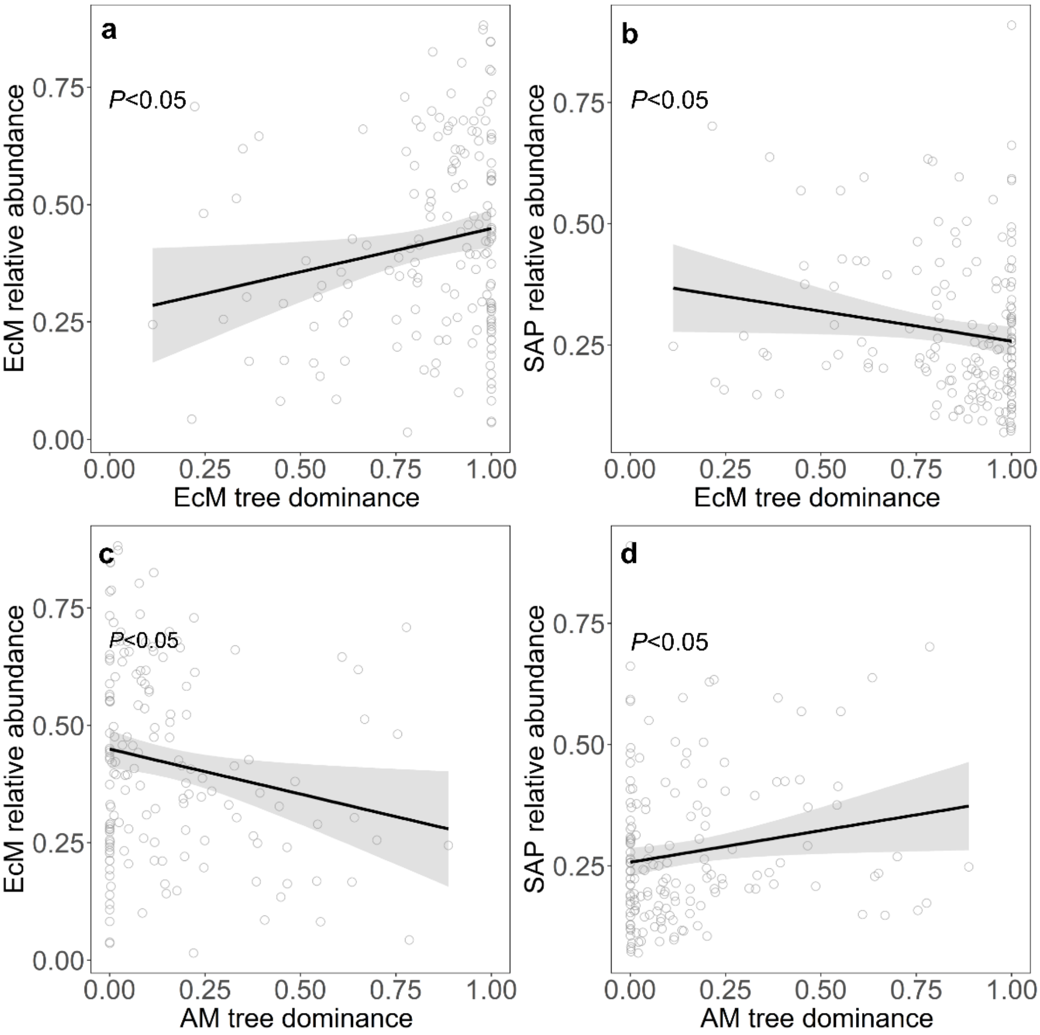

Figure S3 **Relationship between ectomycorrhizal (EcM)/saprophytic (SAP) fungal relative abundance and AM/EcM tree dominance.** a, relationship between EcM relative abundance and EcM tree dominance; b, relationship between SAP fungal relative abundance and EcM tree dominance; c, relationship between EcM relative abundance and AM tree dominance; d, relationship between SAP relative abundance and AM tree dominance. The black line represents simple regression fitted across all forest plots, with gray bands indicating a 95% confidence interval. A solid line indicates $p < 0.05$, while a dotted line represents non significance.

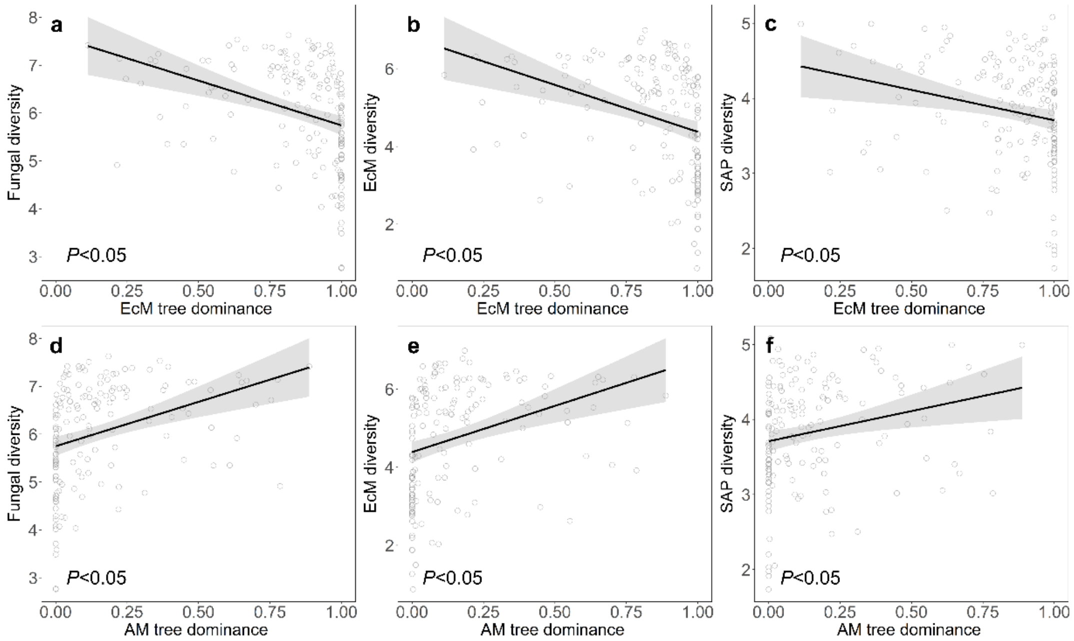

Figure S4 **Relationship between all/EcM/SAP fungal diversity and AM/EcM tree dominance.** The black line represents a simple regression fitted across all forest plots, with gray bands indicating a 95% confidence interval. A solid line indicates $p < 0.05$, while a dotted line represents non-significance.
